# Effects of Warming on CO_2_ Fluxes in an Alpine Meadow Ecosystem on the Central Qinghai–Tibetan Plateau

**DOI:** 10.1371/journal.pone.0132044

**Published:** 2015-07-06

**Authors:** Hasbagan Ganjurjav, Qingzhu Gao, Weina Zhang, Yan Liang, Yawei Li, Xujuan Cao, Yunfan Wan, Yue Li, Luobu Danjiu

**Affiliations:** 1 Institute of Environment and Sustainable Development in Agriculture, Chinese Academy of Agricultural Sciences, Beijing, China; 2 Key Laboratory for Agro-Environment & Climate Change, Ministry of Agriculture, Beijing, China; 3 Clinic Pharmacy of Qinghai Hospital of Traditional Chinese Medicine, Qinghai Province, Xining, China; 4 Nagqu Agriculture and Animal Husbandry Bureau, Tibet Autonomous Region, Nagqu, China; Nanjing University, CHINA

## Abstract

To analyze CO_2_ fluxes under conditions of climate change in an alpine meadow on the central Qinghai–Tibetan Plateau, we simulated the effect of warming using open top chambers (OTCs) from 2012 to 2014. The OTCs increased soil temperature by 1.62°C (*P* < 0.05), but decreased soil moisture (1.38%, *P* < 0.05) during the experiments. The response of ecosystem CO_2_ fluxes to warming was variable, and dependent on the year. Under conditions of warming, mean gross ecosystem productivity (GEP) during the growing season increased significantly in 2012 and 2014 (*P* < 0.05); however, ecosystem respiration (ER) increased substantially only in 2012 (*P* < 0.05). The net ecosystem CO_2_ exchange (NEE) increased marginally in 2012 (*P* = 0.056), did not change in 2013(*P* > 0.05), and increased significantly in 2014 (*P* = 0.034) under conditions of warming. The GEP was more sensitive to climate variations than was the ER, resulting in a large increase in net carbon uptake under warming in the alpine meadow. Under warming, the 3-year averages of GEP, ER, and NEE increased by 19.6%, 15.1%, and 21.1%, respectively. The seasonal dynamic patterns of GEP and NEE, but not ER, were significantly impacted by warming. Aboveground biomass, particularly the graminoid biomass increased significantly under conditions of warming. Soil moisture, soil temperature, and aboveground biomass were the main factors that affected the variation of the ecosystem CO_2_ fluxes. The effect of warming on inter- and intra-annual patterns of ecosystem CO_2_ fluxes and the mechanism of different sensitivities in GEP and ER to warming, require further researched.

## Introduction

Grassland is a widely distributed vegetation type in terrestrial ecosystems, constituting 32% of the vegetated surface area of the earth, and thus representing an important carbon pool [1]. The carbon storage capacity of terrestrial ecosystems is 3-fold higher than that of the atmosphere. The CO_2_ exchange between terrestrial ecosystems and the atmosphere is dependent on the balance between photosynthesis, respiration, and decomposition of organic matter [2]. Recently, it has been predicted that global warming will cause a widespread changes in the carbon balance of terrestrial ecosystems. Moreover, carbon exchange between terrestrial ecosystems and the atmosphere plays an important role not only in the spatial and temporal stability of ecosystems, but also in determining the CO_2_ concentration in the atmosphere, which thereby has a feedback effect on climate change and carbon levels in terrestrial ecosystems [3,4].

Ecosystem CO_2_ fluxes can be represented by the net ecosystem CO_2_ exchange (NEE), defined as the difference between carbon uptake by gross ecosystem productivity (GEP) and carbon release by ecosystem respiration (ER) [2]. It is generally assumed that the optimum temperature for plant photosynthesis is lower than that for respiration, on account of the different temperature sensitivities of the two processes. For example, warming may cause a higher rate of CO_2_ emission than CO_2_ absorption, resulting in a reduction in the vegetation carbon pool [5,6]. However, this assumption is controversial, as the variation of the temperature sensitivities of photosynthesis and respiration vary in different ecosystems [6]. Some studies have indicated that warming has no significant effect [7] or even a positive effect [8] on the NEE. A meta-analysis of 85 global warming experiments (2011) showed that warming can accelerate both photosynthesis and respiration, but has no significant effect on the net carbon uptake of an ecosystem [7]. In an experimental study in an alpine region of the Qinghai—Tibetan Plateau, Peng *et al*. (2014) showed that experimental warming stimulated GEP more than ER, leading to an increase in NEE [8].

In addition to temperature, other factors affecting ecosystem carbon flux values include water availability, plant productivity, community composition, and plant nitrogen content [9]. Under conditions of warming, these biotic and abiotic factors may differentially regulate the balance between GEP and ER, thus influencing the NEE of an ecosystem. For example, grassland productivity closely related to autotrophic respiration and photosynthesis, and indirectly affects heterotrophic respiration in plant roots [6,10]. Thus, warming can have significant but variable impacts on the carbon budget of terrestrial ecosystems by influencing the plant productivity and species composition [11]. It is generally believed that warming has a positive effect on both GEP and ER [12–14]. But under drought conditions, warming clearly inhibits the carbon uptake of ecosystems [4] and also may reduce the carbon emission of grassland ecosystems by limiting soil microbial activity and root respiration [15]. Warming could therefore result in changes in plant productivity, plant community structure, nutrient availability, and soil microclimate [10,16–18]. Considering the variable impacts of these processes on the NEE, predicting the response of NEE to biotic and abiotic factors under conditions of warming presents substantive difficulties [19,20].

The central Qinghai—Tibetan Plateau (also referred to as ‘Changtang’), located to the north of Gangdisê Mountains and the Nyainqêntanglha Mountains, covering an area of approximately 446,000 km². The region is known as the “roof of the world”, as the average elevation of the Changtang is over 4500 m [21]. This region is the source of numerous rivers, and a major herding region in China. In recent years, marked changes have occurred in the climate of the region; in particular, temperatures in the region have increased, more than in the other parts of China [22]. Grassland is the most important natural ecological system and alpine meadow is the most typical ecosystem type in Central Qinghai—Tibetan Plateau [23]. Alpine meadows are extremely fragile and are therefore particularly sensitive to climate change. In addition, studies have shown that alpine meadows store large volumes of carbon, as high rainfall in alpine regions enhances productivity, and low temperatures reduce decomposition rates [24–26]. Therefore, under conditions of climate change and human disturbance, alpine meadows, which are currently carbon sinks, may convert to carbon sources [24–26].

In the present study, we simulated the effect of warming in an alpine meadow ecosystem of central Qinghai—Tibetan Plateau, by using open top chambers (OTCs). Because of the low mean temperature in this region, we hypothesize that warming would positively impact ecosystem CO_2_ exchange by increasing plant biomass. Our goal was to investigate how GEP and ER respond to warming, and how associated change in soil temperature, soil moisture, and aboveground biomass affect the responses of net CO_2_ uptake of an alpine meadow ecosystem in the central Qinghai—Tibetan Plateau.

## Materials and Methods

### Ethics Statement

No specific permits were required for the described field studies and the field studies did not involve endangered or protected species.

### Site description

The research area is located in Nagqu County, Nagqu Prefecture, Tibet Autonomous Region, China (31.441°N, 92.017°E; 4500m a.s.l.). The mean annual temperature is −1.2°C, the annual precipitation is 431.7 mm, and the annual sunshine (total hours) is 2789.9 h (the average values from 1955 to 2011). More than 90% of the annual rainfall in the area occurs during the relatively warm rainy season (May–September) when the average monthly temperature is >0°C. The annual mean temperature has increased by 0.31°C per decade from 1961 to 2008 [19]. The predominant ecosystem in this area is grassland, with alpine meadow being the main grassland type. The sedges *Kobresia pygmaea* and *Carex moorcroftii* and the grass *Poa pratensis* are the main graminoid species, and *Potentilla acaulis* and *Oxytropis ochrocephala* are the main forbs. At the study site, located in an alpine meadow, the average vegetation coverage was >80% and the peak annual biomass was approximately 50 g m^−2^. The soil bulk density was 1.01 g cm^−3^ and the soil pH was 7.05. Organic carbon, total carbon, total nitrogen, and total phosphorus contents of the soil at a depth of 0–15 cm were 41.39, 49.84, 6.78, and 1.43 g kg^−1^, respectively. Details of soil physicochemical properties and soil enzymes are provided in Wang *et al*. (2014) [18].

### Experimental design

We initiated our whole year warming experiment in *K*. *pygmaea* meadow in July 2011, where we established a total of eight experimental plots (four treatment plots and four control plots). The OTCs used in the treatment plots were made of solar transmitting materials and were the shape of a truncated cone, with the height of 0.45 m, the diameter of 1.20 m at ground height, and the diameter of 0.65 m at the maximum height; the OTCs were placed on the soil surface and provided year-round warming to the enclosure.

### Soil temperature and moisture

We used the EM50 data collection systems (Decagon Devices, Inc., NE, USA) to obtain microclimate measurement in each plot. Soil temperature and humidity sensor were placed at a depth of 5 cm in each plot. Data were collected at 30-min intervals throughout the growing seasons from 2012 to 2014.

### Aboveground biomass

We measured the aboveground biomass in mid-August (considered as annual peak aboveground biomass) 2012–2014 using a quadrat sampling approach (the area of quadrat was 0.5×0.5m^2^), and then applied a nondestructive method based on linear regressions between aboveground biomass and vegetation height and cover for each species to estimate the total aboveground biomass in the study area. The same measurements were performed in both the study plots and the calibration plots located adjacent to our experimental site. In mid-August, we recorded the plant species composition, cover, and height of each species in the study plots. The leaves and stems of the plants were cut, sorted, and placed in envelopes. The harvested plants, both leaves and stems, were placed in a drying oven for 30 min at 105°C, after which the temperature was maintained at 70°C until a constant weight was reached; the dried plants were then weighed.

### Ecosystem CO_2_ fluxes

We used a portable photosynthesis system (Li-6400; LI-COR Inc., Lincoln, NE, USA) and the transparent chamber method to measure the NEE and ER of the alpine meadow during the growing seasons in 2012–2014. Prior to the experiment, we placed a base in each area (30 cm × 30 cm) for measurement. On sunny days during the growing season, we conducted measurements at 10:00–12:00 of local time, three times per month. Initially, we placed a transparent polyethylene chamber (30 cm × 30 cm ×40 cm) on the base in each area. Next, we installed a fan on the roof of each chamber, to mix the gases inside and measure the NEE over a period of 90 s. After NEE measurement, we removed the chamber and allowed the air humidity and CO_2_ values to reach ambient levels. Finally, we replaced the chamber on the base, covered it with a shade cloth (inner side black, outer side white), and measured the ER over a period of 90 s. We calculated the GEP from the NEE and ER, by using the equation GEP = NEE + ER. In our study, positive NEE values refer net C uptake by the ecosystem, while negative NEE values represent net C loss from the ecosystem.

### Data analysis

Seasonal mean values used in this study were calculated from the monthly mean values, which were averaged from all measurements obtained in a given month. A repeated measures analysis of variance (RMANOVA) was used to examine the effects of warming, year, and their interactions on seasonal mean ecosystem CO_2_ fluxes. The RMANOVA was also used to examine warming effects on ecosystem CO_2_ fluxes in each growing season in 2012–2014. Stepwise multiple linear regression analyses were used to examine the relationships between ecosystem CO_2_ fluxes and soil temperature, soil moisture, and aboveground biomass. All analyses were conducted using the SPSS (Statistical Package for the Social Sciences) Statistics versions 20.0.

## Results

### Microclimate changes induced by warming

**Fig 1:**
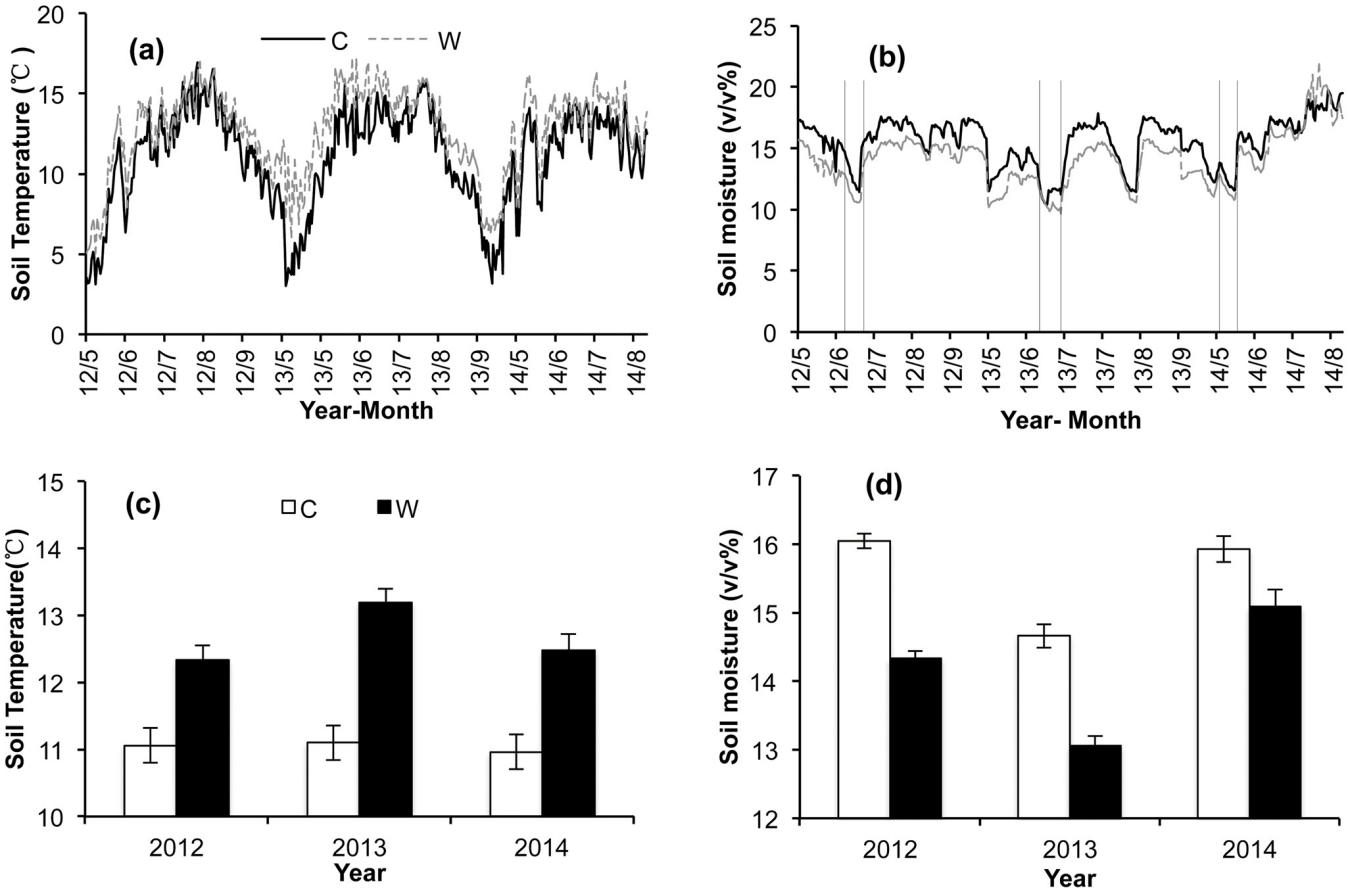
Temporal patterns of soil temperature and soil moisture in control and treatment plots in alpine meadow. C: Control, W: Warming. (a): Intra-annual patterns of soil temperature; (b) Intra-annual patterns of soil moisture; the dotted line indicate the period of spring drought in each year; (c): Inter-annual patterns of soil temperature; (d): Inter-annual patterns of soil moisture.

The microclimate in each plot in the alpine meadow showed similar seasonal patterns during the growing seasons of 2012–2014 (Fig 1a and 1b). During the study period (from May 2012 to September 2014), the daily mean soil temperature in the treatment plots was significantly higher than in the control plot, being higher by 1.27°C, 2.09°C, and 1.52°C during the 2012, 2013, and 2014 growing seasons, respectively. The OTCs increased the daily mean soil temperature by an average of 1.62°C (*P*<0.05, Fig 1c) over the three years. Compared to the control plots, the soil moisture of the treatment plots reduced by 1.71%, 1.61%, and 0.83% in the 2012, 2013, and 2014 growing seasons, respectively. Thus, the average annual decrease in soil moisture caused by the OTCs was 1.38% (*P*<0.05, Fig 1d).

### Effects of warming on CO_2_ fluxes and aboveground biomass

**Fig 2:**
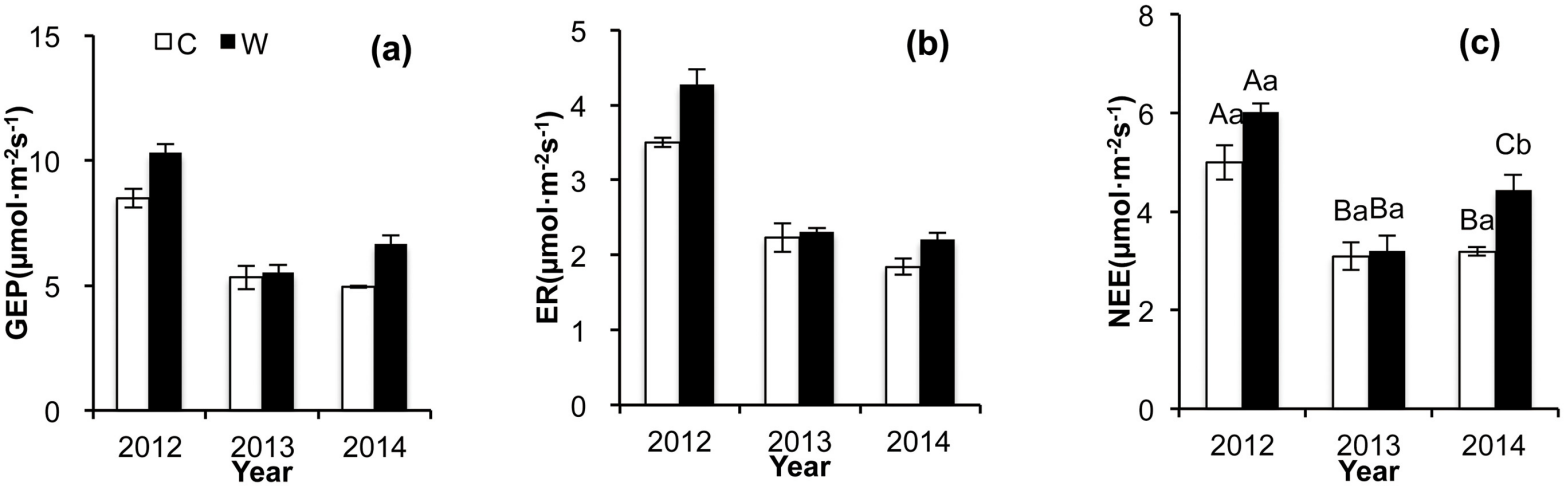
Inter-annual patterns of gross ecosystem productivity (GEP) (a), ecosystem respiration (ER) (b), and net ecosystem CO_2_ exchange (NEE)(c) in control and warming treatments from 2012 to 2014. C: Control, W: Warming. Different capital letters indicated significant different (P<0.05) among years in same treatments; different small letters indicated significant different (P<0.05) among different treatments in same years.

In the treatment plots, as compared to the control plot, the seasonal mean GEP increased by 1.80μmol·m^-2^s^-1^ in 2012 (*P* = 0.028) and 1.70 μmol·m^-2^s^-1^ in 2014 (*P* = 0.024, Fig 2a), and the ER increased by 0.78μmol·m^-2^s^-1^ in 2012 (P = 0.023), showing no change in 2013 and 2014 (*P*>0.05). The greater increases in the GEP values as compared with the ER values resulted in a marginal increase in NEE values in 2012 (*P* = 0.056) and a substantial increase in 2014 (*P* = 0.034, Fig 2b and 2c). Over the 3-year duration of the experiment, average GEP, ER, and NEE in the treatment plots increased by 19.6%, 15.1%, and 21.1%, respectively.

**Fig 3:**
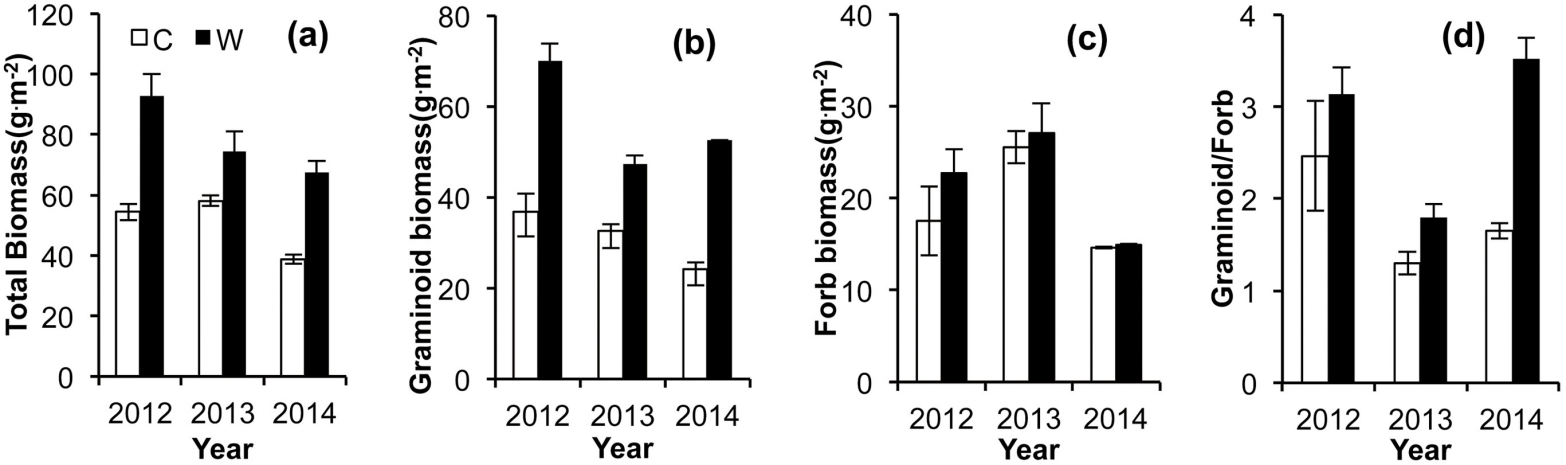
Total aboveground biomass (a), graminoid biomass (b), forb biomass (c), and ratio of graminoid and forb (d) in alpine meadow during 2012–2014 growing seasons. C: Control, W: Warming.

Warming (in the OTCs) significantly increased the aboveground biomass (Fig 3) by 70.7%, 28.1%, and 73.9% in 2012, 2013, and 2014, respectively (Fig 3a, *P*<0.05), and increased the graminoid biomass by 90.0%, 45.4%, and 117.5%, respectively (Fig 3b, *P*<0.05); the forb biomass was not significantly affected by warming (Fig 3c). Therefore, the biomass ratio of graminoids and forbs significantly increased in the warming plots as compared to the control plot (Fig 3d).

### Effects of warming on temporal patterns of CO_2_ fluxes

**Fig 4:**
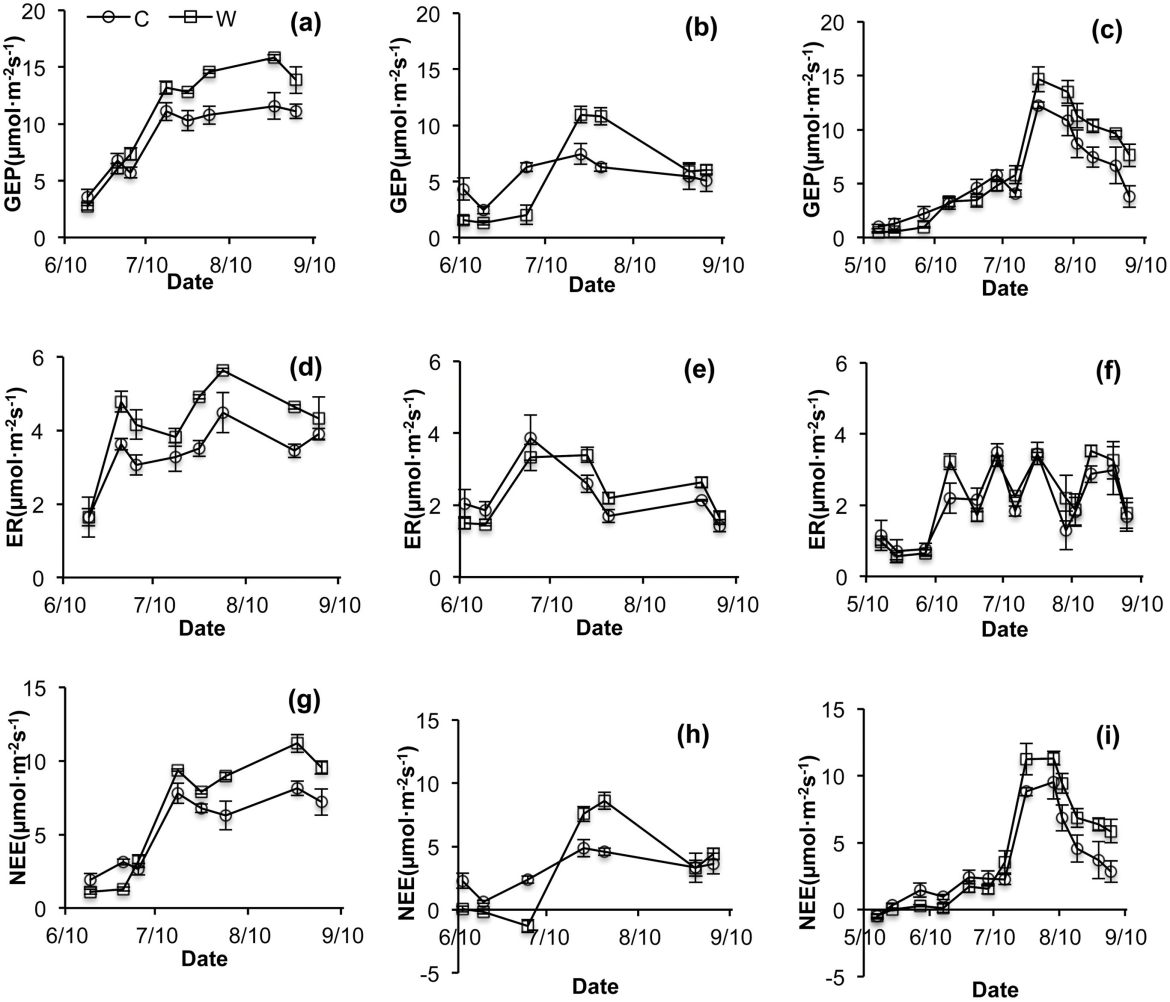
Intra-annual patterns of gross ecosystem productivity (GEP) (a, b, c), ecosystem respiration (ER) (d, e, f), and net ecosystem CO_2_ exchange (NEE) (g, h, i) in 2012 (left panels), 2013 (middle panels) and 2014 (right panels). C: Control, W: Warming.

Results of the RMANOVA indicate significant inter-annual variation in GEP, ER, and NEE values. The GEP, ER, and NEE were all higher in 2012 than in 2013 and 2014 in both control and treatment plots (Fig 2a and 2c). The interaction of warming and year had not a significant effect on GEP, ER, and NEE (*P*>0.05, Table 1). Significant intra-annual variations of CO_2_ fluxes occurred in each year of the experiment (*P*<0.001, Table 2, Fig 4). Intra-annual patterns of the GEP and NEE values were both substantially altered by warming in all the years (*P*<0.05, Table 2, Fig 4). However, the seasonal patterns of ER were not significantly altered under conditions of warming (Fig 4d–4f).

**Table 1:** Results (*P*-values) of repeated measures analysis of variance (RMANOVA) on the effects of warming (W), year (Y), and their interactions on gross ecosystem productivity (GEP), ecosystem respiration (ER), and net ecosystem CO_2_ exchange (NEE).

	GEP	ER	NEE
W	**0.054**	**0.070**	**0.032**
Y	**<0.001**	**<0.001**	**<0.001**
W×Y	0.064	0.073	0.124

**Table 2:** Results (*P*-values) of repeated measures analysis of variance (RMANOVA) on the effects of warming (W), sampling date (D), and their interactions on gross ecosystem productivity (GEP), ecosystem respiration (ER), and net ecosystem CO_2_ exchange (NEE).

	2012			2013			2014		
	GEP	ER	NEE	GEP	ER	NEE	GEP	ER	NEE
W	**0.028**	**0.023**	0.056	0.792	0.741	0.832	**0.024**	0.145	**0.034**
D	**<0.001**	**<0.001**	**<0.001**	**<0.001**	**<0.001**	**<0.001**	**<0.001**	**0.003**	**<0.001**
W×D	**0.043**	0.228	**0.006**	**<0.001**	0.121	**<0.001**	**0.033**	0.205	**0.050**

### Relationships between CO_2_ fluxes and biotic/abiotic factors

**Fig 5:**
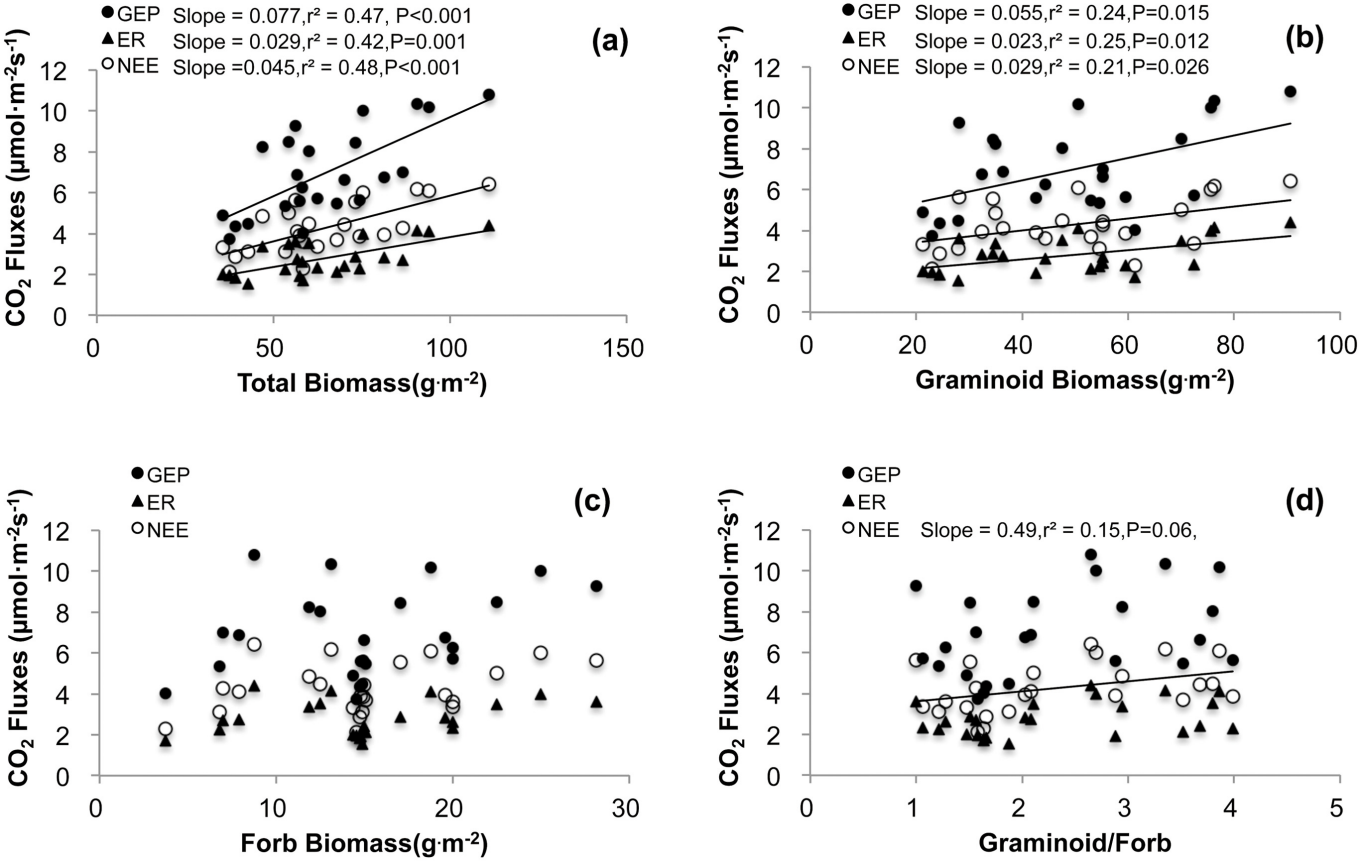
The relationships between ecosystem CO_2_ fluxes: gross ecosystem productivity (GEP) (filled cycles and solid lines), ecosystem respiration (ER) (filled triangles and dashed lines), and net ecosystem CO_2_ exchange (NEE) (open cycles and dotted lines), and aboveground biomass: total biomass, graminoid biomass, forb biomass, and biomass ratio of graminiod/forb across 2012 to 2014.

During the three growing seasons, the temporal variation of GEP, ER, and NEE increased linearly with increasing soil temperature in both the control and warming treatment plots (Table 3). The slope of the regression for GEP—temperature was steeper than ER—temperature in both the control (*P* = 0.022) and warming plots (*P* = 0.071). The soil moisture also influenced the temporal variation of GEP and NEE, but not ER, in both the control and treatment plots (Table 3). Stepwise multiple regression analyses showed that soil temperature and soil moisture together accounted for 49.4% (*P*<0.001) and 42.6% (*P* = 0.001), respectively, of the temporal variation in GEP in the control and warming treatment plots, and were responsible for 50.9% (*P*<0.001) and 44.8% (*P*<0.001), respectively, of the temporal variation in the NEE in the control and warming plots. Moreover, the GEP, ER, and NEE were linearly and positively correlated with the total aboveground biomass (Fig 5a) and graminoid biomass (Fig 5b). The CO_2_ fluxes were not correlated to forb biomass (Fig 5c) and biomass ratio of graminoid and forb (Fig 5d) in the three growing seasons.

**Table 3:** Temporal dependence of gross ecosystem productivity (GEP), ecosystem respiration (ER), and net ecosystem CO_2_ exchange (NEE) on soil moisture and soil temperature across the three growing seasons.

CO_2_ Fluxes	Environmental factors	Plots	Regression equation	*F*	*r* ^*2*^	*P*
**GEP**	Soil Temperature	C	*Y* = 0.74x−2.68	10.56	0.27	**0.003**
		W	*Y* = 1.04x−6.51	6.71	0.19	**0.015**
	Soil Moisture	C	*Y* = 85.78x−7.46	9.97	0.26	**0.004**
		W	*Y* = 67.01x−3.14	10.65	0.28	**0.003**
**ER**	Soil Temperature	C	*Y* = 0.17x + 0.45	4.13	0.13	**0.052**
		W	*Y* = 0.27x−0.79	5.37	0.16	**0.028**
	Soil Moisture	C	*Y* = 13.49x−0.32	1.75	0.06	0.197
		W	*Y* = 5.07x−2.00	0.55	0.02	0.463
**NEE**	Soil Temperature	C	*Y* = 0.60x−3.40	9.78	0.26	**0.004**
		W	*Y* = 2.24x−1.24	5.02	0.15	**0.033**
	Soil Moisture	C	*Y* = 74.97x−8.21	11.69	0.30	**0.002**
		W	*Y* = 61.96x−5.15	14.31	0.34	**0.001**

## Discussion

### Temporal variations of ecosystem CO_2_ fluxes

In the alpine meadow study plots, GEP values during the growing season were higher than ER values, indicating net carbon uptake by vegetation (Fig 2). These results agree with those of previous studies on alpine grasslands in the Qinghai—Tibetan Plateau [8,25,26].

Significant inter- and intra-annual variations of ecosystem CO_2_ fluxes were observed in this study (Figs 2 and 4) were related to variations in soil moisture and soil temperature (Table 3). For example, in 2012, the higher soil moisture levels were associated with resulted in higher CO_2_ fluxes (Figs 1a and 1b and 2). Moreover, levels of aboveground biomass can also affect ecosystem CO_2_ fluxes (Fig 5). Our findings agree with certain other studies of temperate steppes [27], alpine meadows [8], and tundra ecosystems [5]. The GEP was positively correlated with soil moisture and soil temperature. However, the soil moisture could not explain the temporal variation of ER (Table 3). Moreover, the temperature sensitivity of the GEP was significantly higher than the ER (*P* = 0.022, Table 3). These results indicate that GEP is more sensitive to climate variations than ER [8,28].

Warming significantly altered seasonal patterns of the GEP, but did not affect seasonal patterns of the ER, thus resulting in significant changes in the seasonal patterns of NEE in the treatment plots (Table 2, Fig 4). During the early growing seasons, the NEE was lower in the treatment plots than that in the control plots; this pattern was the result of a periodic drought that occurs in late June and early July. After the onset of summer monsoonal rains in mid-July, soil moisture begins to increase, resulting in larger NEE values in the treatment plots than in the control plots during the monsoon season (Figs 1b and 4g–4i). Previous studies have also shown that the effects of warming on ecosystem CO_2_ exchange are modulated mainly by soil moisture [8, 28]. Jiang *et al*. (2012) and Dong *et al*. (2011) both found that the reduction of CO_2_ fluxes in the early growing season was related to spring drought [27,29]. These findings indicate that soil moisture is the main driver of the seasonal variation of CO_2_ fluxes under conditions of warming.

### Effects of warming on ecosystem CO_2_ fluxes

Most previous studies suggest that warming either accelerate carbon loss from grassland ecosystems or have no significant effect [5,13,30]. The results of the present study indicate variable effects of warming on ecosystem CO_2_ fluxes, depending on the year. For example, under warming, the seasonal mean NEE increased slightly in 2012, increased substantially in 2014, but showed no significant change in 2013, as compared with values in control plots. Soil temperature, soil moisture, and aboveground biomass were both correlated with the increase in NEE (Fig 5, Table 3).

In this study, warming resulted in a substantial increase in total aboveground biomass, particularly in graminoid biomass (Fig 3a and 3b). Some studies have shown that shifts in species composition induced by warming can also influence the magnitude of ecosystem CO_2_ fluxes [27,28]. Shi *et al*. (2010) showed that warming had positive effects on photosynthesis in graminoids but negative effects in forbs [31]. These results can be explained in terms of temperature constraints (which are relaxed during warming) on the growth of graminoids and the influence of resource competition (which increases under warming) on the growth of forbs. In 2013, the small response of the NEE (Fig 3) under warming may have been caused by a prolonged spring drought (Fig 1b, width of hashed line) and low mean soil moisture (Fig 1d). Our results are consistent with the finding of those studies on temperate steppe [28,32]. Xia *et al*. (2009) showed that warming led to an increase in grassland biomass, but that this positive effect was offset by the negative effect of decreases soil moisture, resulting in overall insignificant effects on NEE [28]. Other studies have suggested that soil moisture may represent a major limiting factor of carbon uptake in moisture-limited ecosystems, especially in arid and semi-arid environments [33–35]. These observations suggest that under conditions of drought, the response of NEE to warming in alpine meadows is similar to that in temperate steppes.

### Variable responses of GEP and ER to warming

The relative contributions of GEP and ER determine the carbon source/sink capabilities of ecosystems [36]. Law *et al*. (2002) summarized the data from a number of studies on carbon exchange in forest, grassland, farmland, and tundra ecosystems, and demonstrated that the GEP is positively related to temperature [37]. In grassland ecosystems, each biotic and abiotic process, including temperature and moisture, soil microbial activity, species composition, and land use pattern, influences ecosystem carbon output [11,38,39]. Our findings show that under conditions of warming, the increase in GEP was greater than the increase in ER in both 2012 and 2014, resulting in an increase in the net carbon uptake in those years (Fig 2). In two of the three years of this study, we observed no significant effects of warming on ER, and the sensitivity of ER to warming was less than that of GEP (Fig 2, Table 3). These findings are in agreement with those of Xia *et al*. (2009) and Niu *et al*. (2009), who reported minimal effects of warming on ER [28,32]. However, unlike our study, Jiang *et al*. (2012) found that both ER and GEP are strongly sensitivity to climate conditions, and show similar patterns of response [27]. Other studies have also shown that warming can induce a substantial increase in the ER [14,39]. Thus, the response of ER to an increase in temperature may vary in different ecosystems [6].

In this study, the response of net carbon uptake to warming was mainly driven by the temperature-sensitivity of the GEP. The NEE increased significantly when the GEP increased substantially, but did not change when the GEP showed no significant change (Fig 2). The seasonal pattern of the GEP was however modulated by moisture, showing a significant decrease during drought conditions in the early growing season. Therefore, the effects of warming on seasonal mean CO_2_ fluxes in the study area may be modulated by the timing of onset of the summer monsoon (Fig 1b).

## Conclusions

In an alpine meadow on the central Qinghai—Tibetan Plateau, ecosystem CO_2_ fluxes responded positively to warming, with a greater increase in GEP than in ER, resulting in an increase in the net carbon uptake during our 3-year experiment. Warming had significant effects on temporal pattern of GEP, but not ER, leading to significant but seasonally dependent changes in the NEE. Under conditions of warming, the NEE was reduced during drought conditions in early spring. However, starting with the onset of the summer monsoon, the moisture-limiting constraint was relaxed, and the resulting increase in the NEE offset the decrease in the spring. Ecosystem CO_2_ fluxes were correlated with soil temperature, soil moisture, and aboveground biomass. The GEP was more sensitive to warming than was the ER. Further study is required to explore the mechanisms of varying sensitivities of GEP and ER to warming, and to examine the interactions and impacts of warming and spring drought on ecosystem net carbon uptake in alpine meadow.
